# Supplementary material for: A Bayesian game approach for node-based attribution defense against asymmetric information attacks in IoT networks
Source: PLoS One. 2025 Mar 27;20(3):e0316091. doi: 10.1371/journal.pone.0316091 (PMC11949343; doi:10.1371/journal.pone.0316091)
Supplement: Attachment — Submitted filename: Response to Reviewers.pdf [file pone.0316091.s001.pdf]

Dear Editor and Reviewers:

Thank you for the effort in reviewing our manuscript PONE-D-24-27183R1, "A Node-Based Defense Strategy against Attribution Attack in IoT With Asymmetric Information". In this revision, we have made several key improvements based on the reviewers' feedback. First, we enhanced the clarity of the introduction and methodology sections, ensuring the problem statement and the novelty of the NAADBG model are more prominently highlighted. We also expanded the results and analysis sections, correlating the findings more directly with the proposed methodology and addressing potential issues related to asymmetric information scenarios. Additionally, we incorporated comparisons with multiple baseline models to demonstrate the superior performance of our approach. We further revised the language, grammar, and structure throughout the manuscript for improved readability and flow. Lastly, a brief discussion of the challenges in integrating the NAADBG model into existing security infrastructures has been added to the conclusion. These changes aim to address the reviewers' concerns and improve the overall presentation and coherence of the paper.

Yours sincerely,
Jun Chen, Xin Sun, Wen Tian, Guangjie Liu

Response to the Editor

Comment

Please ensure that your manuscript meets PLOS ONE's style requirements, including those for file naming.

Response:

Thank you for your feedback. We have carefully reviewed PLOS ONE's style requirements and ensured that our manuscript now adheres to the journal's guidelines, including proper file naming conventions. All files have been renamed according to the specified standards to ensure compliance. Should there be any further adjustments required, we are happy to make them promptly.

Comment

We note that the grant information you provided in the 'Funding Information' and 'Financial Disclosure' sections do not match.
When you resubmit, please ensure that you provide the correct grant numbers for the awards you received for your study in the 'Funding Information' section.

Response:

Thank you for your note. We have reviewed the discrepancies between the 'Funding Information' and 'Financial Disclosure' sections. Upon resubmission, we will ensure that the correct grant numbers and funding details are provided consistently across both sections. We appreciate your attention to this detail and will carefully align the information to avoid any inconsistencies.

Comment

In the online submission form, you indicated that [Data are available when requested.].
All PLOS journals now require all data underlying the findings described in their manuscript to be freely available to other researchers, either 1. In a public repository, 2. Within the manuscript itself, or 3. Uploaded as supplementary information.
This policy applies to all data except where public deposition would breach compliance with the protocol approved by your research ethics board. If your data cannot be made publicly available for ethical or legal reasons (e.g., public availability would compromise patient privacy), please explain your reasons on resubmission and your exemption request will be escalated for approval.

Response:

Thank you for bringing this to our attention. Upon resubmission, we will clarify that all data underlying the findings described in the manuscript are available **within the manuscript itself.** We will ensure that any necessary data is clearly included in the paper for full compliance with PLOS ONE's data availability policy.

1 Response to reviewer 1

Comment 1.1

Title is needed to relook and make it more appropriate in the view of contribution.

Response:

We have revised the title to better reflect the core contribution of our paper. The new title is: **"A Bayesian Game Approach for Node-Based Attribution Defense Against Asymmetric Information Attacks in IoT Networks."** This title highlights the use of a Bayesian game framework and clarifies the focus on attribution defense in the context of IoT networks under asymmetric information conditions.

Comment 1.2

The paper's abstract should focus on the importance of addressing this issue. The highlights of the results and how the reader might benefit from the paper's material should also be included.

Response:

We have significantly revised the abstract to better emphasize the importance of addressing attribution attacks in IoT networks, particularly under asymmetric information. The revised abstract also highlights the main results of our work, including the development of a Mixed Strategy Bayesian Nash Equilibrium (MSBNE) for defense strategy selection and the practical implications of our findings. The revised abstract now clarifies how readers and practitioners can benefit from the methodology and results presented in this paper. In the revised version, we revise as follows:

In the rapidly evolving landscape of the Internet of Things (IoT), traditional defense mechanisms struggle to counter sophisticated attribution attacks, especially under asymmetric information conditions. This paper introduces a novel Bayesian game framework—the Node-Based Attribution Attack-Defense Bayesian Game (NAADBG) Model—to address these challenges in IoT networks. The model incorporates a comprehensive set of attacker and defender profiles, capturing the complexities of real-world security scenarios. We develop a refined method for quantifying the payoffs of node-level attack-defense actions and explore the existence of a Mixed Strategy Bayesian Nash Equilibrium (MSBNE), enabling optimal defense strategy selection. Our simulations demonstrate that the NAADBG model significantly enhances network defense performance by optimizing resource allocation and preempting potential threats. This approach provides critical insights into developing proactive defense strategies against attribution attacks, contributing to more resilient IoT security frameworks. The results show that this method not only improves network defense performance but also presents practical applications in strengthening real-time IoT environments.

Comment 1.3

The Introduction section needs to relook to improve its quality and readability.

Response:

We have revised the introduction to enhance both its quality and readability. The updated introduction now clearly outlines the key challenges in defending IoT nodes from attribution attacks, particularly under asymmetric information. We have also restructured the text to focus more on the research problem and the contributions of our paper, ensuring that readers can easily grasp the importance and innovation of the proposed solution.

Comment 1.4

Further, it is suggested to add an RELATED STUDY Section and need to add the existing work and make an comparative table to address the various key gaps in existing work which are going to address through this paper.
In today's increasingly interconnected world, the security of IoT networks has become a significant challenge, particularly at the node level, where individual devices are vulnerable to advanced and evolving cyber threats [1,2]. The complexity of protecting billions of IoT devices from attribution attacks—where attackers conceal their identity and origin—has exposed critical vulnerabilities in traditional defense mechanisms. This paper focuses on the novel challenge of defending IoT nodes from attribution attacks in an asymmetric information environment, where defenders have limited knowledge of attackers' strategies [3]. Addressing these vulnerabilities is crucial for maintaining the integrity of IoT networks, as compromised nodes can lead to severe consequences, including data theft, privacy violations, and large-scale network disruptions.
Traditional defense mechanisms, though effective in many areas, struggle to keep pace with the rapid development of attribution attack methods [4,5]. These methods, which often involve obfuscating attack sources and exploiting gaps in network defenses, present a serious challenge, especially in environments where defenders lack full knowledge of the attackers' strategies. Attackers can exploit this information asymmetry to their advantage, launching sophisticated attacks while remaining undetected. Therefore, it is imperative to develop defense mechanisms that can anticipate such threats, even with incomplete information.
Cyber adversaries frequently target IoT nodes, seeking to steal sensitive information such as financial data, personal records, or proprietary business information. In addition, attackers may compromise IoT components like edge nodes to orchestrate larger, more coordinated attacks. These threats highlight the necessity of defense strategies that go beyond reacting to attacks and proactively mitigate risks in environments where defenders are at an information disadvantage [6,7]. Furthermore, traditional defenses that rely on predefined rules or signature-based detection are often insufficient against zero-day attacks and advanced persistent threats. The ever-growing complexity of modern network environments makes it even more challenging to maintain up-to-date and comprehensive security measures. To address these challenges, this paper introduces a Node-Based Attribution Attack-Defense Bayesian Game (NAADBG) model, which incorporates a game-theoretic approach to model the interactions between attackers and defenders in an asymmetric information setting. The NAADBG model enables defenders to anticipate attacker moves, assess the risks, and strategically allocate limited resources to enhance the defense of IoT nodes. The model not only considers the behaviors and strategies of both attackers and defenders but also accounts for the uncertainties present in real-world network scenarios. The main contributions are as follows:

- We propose a novel game-theoretic model tailored to IoT networks that addresses the problem of attribution attacks in asymmetric information environments. The model NAADBG incorporates both attacker and defender profiles to enhance its applicability to real-world security scenarios.
- We refine the quantification of payoffs for both attackers and defenders by assessing the impact of node-level attack-defense actions. We analyze the existence of a Mixed Strategy Bayesian Nash Equilibrium (MSBNE) and derive an optimal defense strategy selection method.
- Through simulation experiments, we evaluate the performance of the proposed model. The results demonstrate that the NAADBG model significantly improves network defense by optimizing resource allocation and preemptively mitigating threats.

Response:

We have added a "Related Work" section, which reviews key studies on the application of game theory in cybersecurity. A comparative table has been included to highlight the gaps in existing research, particularly in relation to attribution attacks and information asymmetry. Our paper addresses these gaps by proposing the Node-Based Attribution Attack-Defense Bayesian Game (NAADBG) Model, which is specifically designed to handle IoT network security under asymmetric information conditions. This comparative table clarifies how our contributions build on and extend the current state of the art.

We have also emphasized the novelty of our Node-Based Attribution Attack-Defense Bayesian Game (NAADBG) model, which addresses the issue of information asymmetry in IoT networks, a gap not sufficiently covered in previous literature.

Comment 1.5

Performance reviews are mostly used to summarize the findings of interpretation and inference, highlighting new discoveries and innovations. So need to be validate in respect to the obtained results.

Response: Thank you for your valuable feedback. We have revisited the performance analysis section to ensure that the

Table 1: Comparison of Related Work in Game-Theoretic Cybersecurity

Author(s)	Focus	Contribution	Limitations
Do et al. (2017) [14]	Game theory in security and privacy	Review of cyber-physical and communication security	Limited discussion on attribution
Liang and Xiao (2013) [15]	Cooperative/non-cooperative games	Game theory in network defense	Assumes symmetric information
Manshaei et al. (2013) [16]	Game theory in network security	Strategic decision-making in cyber defense	Lacks focus on real-world asymmetry
Iqbal et al. (2019) [17]	Cybersecurity modeling challenges	Discusses complexity in game models	Limited on anti-attribution strategies
Zhang et al. (2022) [10]	Anti-attribution in IoT	Advanced anti-attribution techniques	No game-theory modeling

findings and interpretations are closely aligned with the obtained results. In the revised manuscript, we have provided a more detailed discussion of the simulation outcomes, directly linking them to the proposed Node-based Attribution Attack-Defense Bayesian Game (NAADBG) model. We have also included a comparison with baseline methods such as Traditional Game Theory, Cooperative Game Theory, and the Hybrid Game Model, to further validate the innovations and effectiveness of the NAADBG model in improving defense payoffs, especially as the defense success rate (β) increases. This updated analysis clearly highlights the innovations introduced by the NAADBG model and its superior performance in handling complex, evolving threats in IoT environments.

Interestingly, the payoffs for attackers remain stable as $B(A_h)$ increases, especially for ADA and AEA strategies without active attacks (NA), maintaining consistent values. This suggests that while the cost of executing attacks increases, it does not significantly impact the attackers' immediate payoffs, highlighting the importance of balancing defense resource allocation against different attack costs.

Fig. 6 examines how the attack impact, $W(C_x)$, affects the payoffs in NAADBG. As $W(C_x)$ increases, the payoffs for senior defenders (SD) rise steadily from negative values to over 1100, indicating that defense becomes more effective as the potential attack impact increases. Senior defenders who do not employ active defense (ND) maintain consistent payoffs, suggesting that sophisticated defense strategies can still mitigate attack impacts without active intervention.

On the other hand, both ADA and AEA strategies see rising payoffs as $W(C_x)$ grows, reflecting the increasing potential rewards for successful attacks. This highlights the need for defenders to adjust their strategies based on the potential impact of attacks, ensuring that high-value targets receive more robust protection to counter attackers' increased motivations.

We compare with three baseline methods:

- Traditional Game Theory [14]: Assumes complete information and static strategies, limiting its adaptability to advanced threats.
- Cooperative Game Theory [15]: Focuses on resource sharing among defenders, which enhances defense but relies on cooperation.
- Hybrid Game Model [20]: Combines cooperative and non-cooperative approaches, offering flexibility but struggling with complex, asymmetric information scenarios.

Based on the baseline comparison, it is evident that the NAADBG model consistently outperforms other methods, including Traditional Game Theory, Cooperative Game Theory, and the Hybrid Game Model. The NAADBG model demonstrates a sharp increase in defense payoffs as the defense success rate (β) rises, indicating its superior efficiency in resource allocation and adaptability to evolving threats in IoT environments. In contrast, other models show a more gradual and linear increase in payoffs, suggesting that they are less effective at handling complex node-level attribution attacks. The NAADBG's ability to anticipate and counteract sophisticated attacks with limited information makes it the most robust model for optimizing defense strategies.

Comment 1.6

Further the methodology need to be validate in accordance with the used Algorithm Optimal defense strategy selection algorithm based on attack defense game.

Response:

We appreciate your insightful suggestion. In response, we have carefully re-evaluated the methodology section to ensure alignment with the proposed Optimal Defense Strategy Selection Algorithm based on the attack-defense game. We have expanded the explanation of how the algorithm operates within the Node-based Attribution Attack-Defense Bayesian Game (NAADBG) framework, ensuring that the selection process for the optimal defense strategy is clearly justified. Additionally, the simulation experiments have been refined to validate the effectiveness of the algorithm. The results now demonstrate how the algorithm optimizes defense strategies under different attack scenarios, providing quantitative evidence of its effectiveness. These updates ensure that the methodology is robust and well-supported by the experimental outcomes.

To ensure the methodology aligns with the proposed optimal defense strategy algorithm based on the attack-defense game, we have validated the algorithm's core structure and performance. The NAADBG model integrates both attack and defense actions into a Bayesian game framework, enabling the optimal strategy selection for defenders facing node-level attacks in IoT environments. The algorithm computes the mixed strategy Bayesian Nash equilibrium and ranks defense strategies based on their effectiveness in response to varying attacker profiles. Below, we outline the specific aspects of the validation.

Based on the quantification of attack-defense payoffs, the defender's strategy selection is determined by solving for the mixed strategy Bayesian Nash equilibrium. The defense effectiveness is quantified considering the defender's prior beliefs and the payoff matrix. The optimal defense strategy is then selected based on the criterion of maximum defense effectiveness, differentiating this approach from classical algorithms that rely on mixed strategies. Our model advocates for the selection of a pure strategy, which enhances practical operability. By selecting the optimal strategy beforehand, the network is proactively defended against potential security threats. The key steps of this novel defense strategy include: (1) quantifying payoffs for each potential defense strategy; (2) solving for the Bayesian Nash equilibrium to ascertain feasible strategies; (3) evaluating the effectiveness of each strategy based on the equilibrium analysis; and (4) implementing the defense strategy with the highest effectiveness to activate the network's defenses prior to the occurrence of security threats.

Comment 1.7

Additionally, I would advise the authors to verify their work for consistency in grammatical usage, formatting, and reference style

Response:

We appreciate the reviewer's suggestion regarding consistency in grammatical usage, formatting, and reference style. We have thoroughly reviewed the manuscript to ensure that all grammatical issues have been addressed, the formatting is consistent throughout, and the reference style adheres to the journal's guidelines. All necessary corrections have been implemented to enhance the overall clarity and presentation of the paper.

Comment 1.8

Need to relook the Efficiency of our Method section in accordance with the obtained results.

Response:

The efficiency of the proposed Node-based Attribution Attack-Defense Bayesian Game (NAADBG) Model is validated through the results obtained in multiple simulations. These simulations show that our model optimizes defense resource allocation while effectively countering various attacker profiles, such as those focused on attribution depth or efficiency. Specifically, the NAADBG model demonstrates a significant increase in defense success rates (up to 25%) compared to traditional and cooperative game models. This is particularly evident in the resource-limited scenarios, where our method strategically allocates defense measures, resulting in an 18% reduction in resource wastage compared to other models.

Moreover, the NAADBG model shows superior adaptability in scenarios involving asymmetric information, significantly improving the network's overall security metrics. As demonstrated in Figures 2 and 3, the method dynamically adjusts defense strategies based on evolving attack patterns, further contributing to its efficiency in real-world IoT environments. This efficiency is also reflected in the lower computational overhead and faster convergence to Nash Equilibrium compared to baseline methods, as outlined in the complexity analysis.

Comment 1.9

Need to relook the result and analysis section and correlate with the proposed research methodology

Response:

We appreciate the reviewer's suggestion to re-examine the Results and Analysis section. In response, we have revised the section to ensure a closer alignment with the proposed research methodology. The updated analysis now explicitly correlates the key results, such as the impact of defense success rate and resource allocation, with the methodology described earlier in the paper. We have emphasized how the mixed-strategy Bayesian Nash Equilibrium and payoff quantification directly contribute to the obtained outcomes. We believe this revised section better demonstrates the effectiveness of our approach and addresses the reviewer's concerns.

Interestingly, the payoffs for attackers remain stable as $B(A_h)$ increases, especially for ADA and AEA strategies without active attacks (NA), maintaining consistent values. This suggests that while the cost of executing attacks increases, it does not significantly impact the attackers' immediate payoffs, highlighting the importance of balancing defense resource allocation against different attack costs.

Fig. 6 examines how the attack impact, $W(C_x)$, affects the payoffs in NAADBG. As $W(C_x)$ increases, the payoffs for senior defenders (SD) rise steadily from negative values to over 1100, indicating that defense becomes more effective as the potential attack impact increases. Senior defenders who do not employ active defense (ND) maintain consistent payoffs, suggesting that sophisticated defense strategies can still mitigate attack impacts without active intervention.

On the other hand, both ADA and AEA strategies see rising payoffs as $W(C_x)$ grows, reflecting the increasing potential rewards for successful attacks. This highlights the need for defenders to adjust their strategies based on the potential impact of attacks, ensuring that high-value targets receive more robust protection to counter attackers' increased motivations.

We compare with three baseline methods:

- Traditional Game Theory [14]: Assumes complete information and static strategies, limiting its adaptability to advanced threats.
- Cooperative Game Theory [15]: Focuses on resource sharing among defenders, which enhances defense but relies on cooperation.
- Hybrid Game Model [20]: Combines cooperative and non-cooperative approaches, offering flexibility but struggling with complex, asymmetric information scenarios.

Based on the baseline comparison, it is evident that the NAADBG model consistently outperforms other methods, including Traditional Game Theory, Cooperative Game Theory, and the Hybrid Game Model. The NAADBG model demonstrates a sharp increase in defense payoffs as the defense success rate (β) rises, indicating its superior efficiency in resource allocation and adaptability to evolving threats in IoT environments. In contrast, other models show a more gradual and linear increase in payoffs, suggesting that they are less effective at handling complex node-level attribution attacks. The NAADBG's ability to anticipate and counteract sophisticated attacks with limited information makes it the most robust model for optimizing defense strategies.

Comment 1.10

Need to relook on English, figure quality, equations and connectivity among all sections. The overall manuscript should be checked for typos, syntax, and grammar to improve the quality of content flow and presentation.

Response:

We appreciate the reviewer's comments regarding the quality of English, figure quality, equations, and overall connectivity among sections. In response, we have conducted a thorough revision of the manuscript with the following improvements:

- English Language Review: We have carefully revised the entire manuscript for grammatical accuracy, improved syntax, and readability. The content now flows more smoothly, ensuring clearer communication of the research findings.
- Figure Quality Enhancement: All figures have been checked for clarity, resolution, and labeling. We have ensured that the figures are well-integrated into the text and accurately represent the discussed results.
- Equation Consistency: We reviewed all equations for formatting and consistency. Each equation has been appropriately numbered, and its notation has been made consistent throughout the manuscript.
- Improved Section Connectivity: We have revised the transitions between sections to ensure smooth content flow and clear logical progression. Each section now reinforces the narrative of the research more cohesively.

Additionally, we performed a final proofreading of the entire manuscript to address any remaining typos or formatting issues. We believe these revisions significantly improve the quality of the manuscript and address the reviewer's concerns.

2 Response to reviewer 2

Comment 2.1

Briefly describe any existing models and highlight what qualities distinct your proposed NAADBG model. What are the key innovations or improvements your model offers?

Response:

We appreciate the reviewer's request for a clearer comparison between the existing models and our proposed NAADBG model, as well as the need to highlight the distinct qualities and innovations of our approach. In the revised manuscript, we have added a section that outlines the key differences between traditional models such as Traditional Game Theory, Cooperative Game Theory, and Hybrid Game Models.

Here is a brief description of the enhancements our NAADBG (Node-based Attribution Attack-Defense Bayesian Game) model offers:

- Asymmetric Information Handling: Traditional models often assume symmetric information, which is not reflective of real-world IoT security scenarios where defenders usually have limited knowledge about attacker strategies. The NAADBG model specifically addresses this gap by incorporating information asymmetry, allowing it to model more realistic interactions.
- Bayesian Nash Equilibrium (BNE) Framework: The NAADBG model leverages Bayesian game theory to account for incomplete information and calculate the optimal defense strategy using mixed strategy Nash equilibrium. This is a significant improvement over models that rely solely on predefined attack and defense strategies.
- Node-Level Defense: Our model focuses on the node level in IoT networks, making it highly adaptable to scenarios involving highly distributed devices. Many existing models focus on broader network defense without addressing the vulnerabilities at individual node levels, which is critical in IoT environments.
- Strategic Resource Allocation: The NAADBG model is designed to optimize the allocation of defense resources based on real-time data and probabilistic outcomes, unlike some static models that cannot adjust defense strategies dynamically.

These innovations enable our model to outperform the baselines in terms of adaptability, efficiency, and its ability to anticipate complex attacks. This is reflected in our simulation results, where the NAADBG shows a more significant increase in defense payoffs compared to other models.

We believe these additions and explanations address the reviewer's concerns and provide a clearer understanding of how our NAADBG model distinguishes itself from existing work.

Comment 2.2

Given that the model assumes all participants aim to optimize their outcomes, how does the model perform if attackers fail to achieve their optimal outcomes, especially in scenarios involving asymmetric information?

Response:

The NAADBG (Node-based Attribution Attack-Defense Bayesian Game) model is built on the assumption that both attackers and defenders strive to optimize their outcomes in a scenario of asymmetric information. However, it is crucial to explore what happens if attackers do not achieve their optimal outcomes, particularly in situations where their strategies are influenced by incomplete information.

In cases where attackers fail to achieve their optimal outcomes, several important dynamics unfold within the model:

- Suboptimal Attacker Strategies: Attackers' failure to optimize results in reduced payoffs, potentially leading to adjustments in their strategies. The NAADBG model accounts for this through probabilistic approaches (via Bayesian Nash Equilibrium), enabling both sides to adapt based on prior beliefs about their opponent's actions.
- Defender Advantage: When attackers make suboptimal moves, defenders can exploit these weaknesses. The model allows defenders to dynamically adjust their strategies, focusing on areas that attackers under-target or miscalculate due to the information asymmetry.
- Resilience to Information Asymmetry: The model thrives in asymmetric information scenarios, where attackers may struggle to optimize their actions. The NAADBG model continuously updates the defender's beliefs and strategies, maintaining a robust defense even if attackers fail to reach their optimal outcomes.

After all, when attackers perform suboptimally, NAADBG allows defenders to adapt effectively, maintaining an advantage and ensuring resilience in the face of asymmetric information. In response to this concern, we have expanded the original manuscript to include a more detailed discussion of scenarios where attackers fail to achieve their optimal outcomes due to asymmetric information.

Note that in scenarios where attackers fail to achieve their optimal outcomes, particularly under asymmetric information, the NAADBG model dynamically adjusts the defender's strategies by leveraging Bayesian updating. As the attacker deviates from optimal behavior, the defender revises their prior beliefs about the attacker's strategy, enabling more accurate predictions of future actions. This adaptability ensures that even in cases of suboptimal attacker actions, the model remains effective at selecting defense strategies that maximize network protection.

Comment 2.3

Nash Equilibrium has its limitations, such as the presence of multiple equilibria, the possibility of inefficient outcomes, and assumptions about rationality and static interactions. Please further address the limitations of your model.

Response:

We appreciate the reviewer's insightful comment regarding the limitations of Nash Equilibrium and its application in our model. We have added a discussion section in the revised manuscript to address these points in detail.

Firstly, we acknowledge that the presence of multiple equilibria can introduce ambiguity when selecting optimal strategies. To mitigate this, future research could consider refinement methods like selecting focal points or trembling-hand perfect equilibrium, as noted in the manuscript.

Secondly, we agree that Nash Equilibrium does not always guarantee efficient outcomes. This limitation, wherein the strategies may lead to suboptimal resource allocation, has been highlighted in the revised version. We suggest that further work could explore Pareto efficiency or mechanism design to improve resource distribution.

Thirdly, the assumption of rational behavior is indeed an idealized condition, and we have now explicitly mentioned in the manuscript that real-world decision-making often involves bounded rationality. Future iterations of our model could include these aspects to better reflect realistic cybersecurity dynamics.

Finally, the static nature of Nash Equilibrium assumes a one-time interaction between attackers and defenders. We have included a note in the revised text discussing potential future extensions using dynamic game-theoretic models, such as stochastic games or Markov decision processes, to address this limitation.

We believe these additions provide a clearer understanding of the scope and limitations of our model, and we hope this addresses the reviewer's concerns effectively.

Comment 2.4

Briefly describe any potential challenges of integrating this new model into existing security infrastructure and compatibilities.

Response:

We appreciate the reviewer's suggestion to address potential challenges in integrating our proposed NAADBG model into existing security infrastructures. In the revised manuscript, we have added a discussion in the conclusion section highlighting these challenges.

One of the main challenges is the complexity of implementing a Bayesian game model that relies on information asymmetry within current security architectures. Traditional security systems often rely on predefined rules and signature-based approaches, which may not be immediately compatible with a dynamic, game-theoretic strategy. Integrating the NAADBG model would require significant adjustments to the existing detection and response mechanisms, such as incorporating advanced monitoring systems capable of gathering and processing real-time data to model attacker behaviors.

Another challenge is the computational overhead associated with calculating and updating Nash equilibria in real-time. Current security infrastructures may lack the resources to handle these calculations efficiently, particularly in large-scale IoT environments with numerous interconnected devices. The implementation of such a model would require optimization strategies to reduce computational load or the use of cloud-based resources to support the processing demands.

Additionally, we have discussed in the conclusion that the successful deployment of the NAADBG model would require compatibility with existing security protocols and standards, as well as training for security personnel to manage the system. The need for cooperation between various layers of infrastructure and stakeholders would also be a critical consideration.

These points have been integrated into the conclusion to provide a clearer perspective on the potential challenges and necessary steps for practical implementation of the NAADBG model.

This paper introduces a Node-based Attribution Attack-Defense Bayesian Game (NAADBG) model to address attribution attacks in IoT environments under information asymmetry. By incorporating diverse attacker and defender profiles, the model closely aligns with real-world network security challenges. We enhance payoff quantification, analyze the existence of mixed strategy Bayesian Nash equilibrium, and derive an optimal defense strategy selection method. Simulation results demonstrate the model's effectiveness in improving defense performance, particularly in resource-constrained environments. However, integrating the NAADBG model into existing security infrastructures may pose challenges, such as computational overhead and compatibility with current systems, which require further research for practical deployment.
